# In-chair movements: Categorizations and patterns over time based on a literature review

**DOI:** 10.1177/10519815251394878

**Published:** 2025-11-19

**Authors:** Aernout Kruithof, Yu (Wolf) Song, Peter Vink

**Affiliations:** 1 Faculty of Industrial Design Engineering, Delft University of Technology, Delft, The Netherlands; 2 Hanze University of Applied Sciences, Industrieel Product Ontwerpen, Groningen, The Netherlands

**Keywords:** seated posture, fidgets, ICM strategies, postural adjustment

## Abstract

**Background:**

In recent years in-chair movements (ICM) have gained attention in comfort and discomfort studies, but the role of these movements in preventing and/or alleviating discomfort remains unclear. Furthermore, differences in study design and terminology make cross-study comparisons difficult.

**Objective:**

This study aims to synthesize current research on ICM, particularly the categorization of different ICM types. It also aims to provide an overview of ICM over time, focusing on their progressions, characteristics, and possible patterns.

**Methods:**

A systematic literature search was conducted based on the PRISMA framework using Scopus, PubMed, and Web of Science databases. Data from the included studies were extracted and organized according to three ICM descriptors: frequency, amplitude, and posture change.

**Results:**

Eighteen out of 230 identified papers met the inclusion criteria. Substantial heterogeneity in terminology and measurement partly explains inconsistencies in findings. Across most studies, ICM frequency increased over time, although a minority reported decreased movement or a “stiffening effect”. Findings regarding ICM amplitude were inconsistent, while a shift or change toward more slumped posture appears to be especially common during driving activities. These variations suggest that ICM patterns are influenced by task demands, seat characteristics, and individual differences.

**Conclusion:**

ICM patterns are not solely time-dependent but are shaped by seat characteristics, task demands, and individual factors. While several studies suggest correlations between ICM strategies and discomfort, the underlying mechanisms remain unclear. Developing a comprehensive ICM framework that integrates movement strategies, and active or dynamic seating approaches will benefit cross-study comparability and provide directions for future ICM research.

## Introduction

Sitting might lead to discomfort.^1^ Following the comfort model from Vink & Hallbeck, occupants might change their posture to prevent or cope with discomfort.^2^ However, much is unknown about this relationship between in-chair movements (ICM) and (dis)comfort.^3^

Studies have indicated that ICM might be a way of managing or coping with perceived discomfort.^1^^4–9^ For instance, Bhatnager et al. (1985),^10^ and Karwowski et al.^11^ proposed that ICM may be a sign that the person is “trying to alleviate musculoskeletal discomfort”, and the number of ICM are shown to increase with fatigue.^12^ Thus, ICM could be considered as a measure of discomfort. Yet, not all studies have established such a correlation between fatigue and ICM,^13^ and some studies have argued that ICM may serve as a tool to prevent discomfort, such as through exercising.^14^ This “two-fold” relation between ICM and discomfort was also recognized in earlier literature reviews.^15^

Despite this attention to ICM, recent papers have acknowledged that categorizations of ICM are “undecided”.^3^ These differences in categorizations create additional difficulties in comparing studies. Furthermore, due to different thresholds, measurements, and study setups, as acknowledged in the review of Hiemstra-van Mastrigt et al.,^15^ there is no consistently established correlation.

This paper is an inquiry aiming to provide an overview of ICM categories that are distinguished in existing literature as well as aiming to create an overview of ICM development over sitting duration, in terms of their progression, characteristics, and possible patterns. It might contribute to a more common understanding of ICM types and patterns which would be constructive in the search for possible relationships with factors of discomfort.

## Method

A systematic literature search was performed based on the PRISMA framework. Included databases were Scopus, PubMed, and Web of Science. The search terms were organized in the following five groups, taking both English and American spelling into account, see Table 1.

**Table 1.** Search terms.

Category	Terms
#1: (Dis)comfort	comfort OR discomfort OR fatigue
#2: Seating	seat* OR sit OR sitting OR chair
#3: Industry Context	aircraft OR airplane OR vehicle OR train OR car OR riding OR drive* OR driving OR rail OR office OR desk
#4: Body Movement/Posture	“postur* n15 change” OR “body n15 movement” OR “seat n15 movement” OR “postural behave*” OR “postural behaviour” OR “sitting behavi” OR “sitting behaviour” OR “postural movement” OR “in-chair movement” OR “chair n15 movement” OR “active seat*” OR “seat* n15 exercis” OR “seat* n15 game” OR “muscle activity” OR musculo-skeletal
#5: Exclusion	heating OR cooling OR ventilation OR “virtual prototyping” OR “digital human modelling” OR patient OR elderly OR child* OR VR OR vision OR agriculture

Search terms were applied to the study title and abstract, using Boolean “AND” operator between categories. The fifth set of terms, “exclusions”, was not used in the PubMed search string due to the narrow scope of this database. Database searches were performed on November 12^th^, 2022. Inclusion criteria were: 1) The study involves real humans as subjects; 2) The study focuses on everyday sitting contexts, e.g., occupational or transit (office environment, car, bus etc.); 3) The study concerns in-chair movements, “sitting posture-changes” over a certain sitting period, or similarly regards movements while sitting as a factor in the study; 4) The paper/study describes or contains an empirical element, reporting empirical results. For this paper, describing ICM over time was added as an inclusion criterion. Screening was performed on title and abstract, and eligibility was assessed after full text reading.

Data from the included studies were compiled using a standardized form in Microsoft Excel. The extraction table captured general study information, reported measures of in-chair movement (ICM), and three primary descriptors used to structure comparisons of ICM: frequency, amplitude, and posture change.

These descriptors were not derived from an existing framework, as no such standardized classification currently exists in the ICM literature.^3^ Instead, they were identified through an iterative review of terminology across the included papers and represent the most commonly reported and conceptually distinct aspects of ICM. Specifically, ICM frequency refers to metrics that quantify the rate or number of ICM events over time, and ICM amplitude encompasses measures reflecting the magnitude or intensity of movement. Posture change denotes substantial shifts in body position, described in the literature with terms such as “posture shifts,” “leaning forward,” “slumping,” or “distinct posture change.”

To ensure analytical consistency, study-specific labels and metrics were mapped onto one of these three descriptors, with categorization decisions reviewed and refined in discussion among the authors. The reported measures and labels included in the extraction table were then used both to review the labels or ‘terminology’ employed across studies to describe ICM and to compare findings according to time-related ICM patterns. Regarding time effects, the table was further structured with sub-columns distinguishing between overall time effects (e.g., regression analyses) and findings related to specific time intervals (e.g., post hoc tests). Data extraction was performed by the first author and discussed with the co-authors until consensus was reached.

## Results

**Figure 1.**
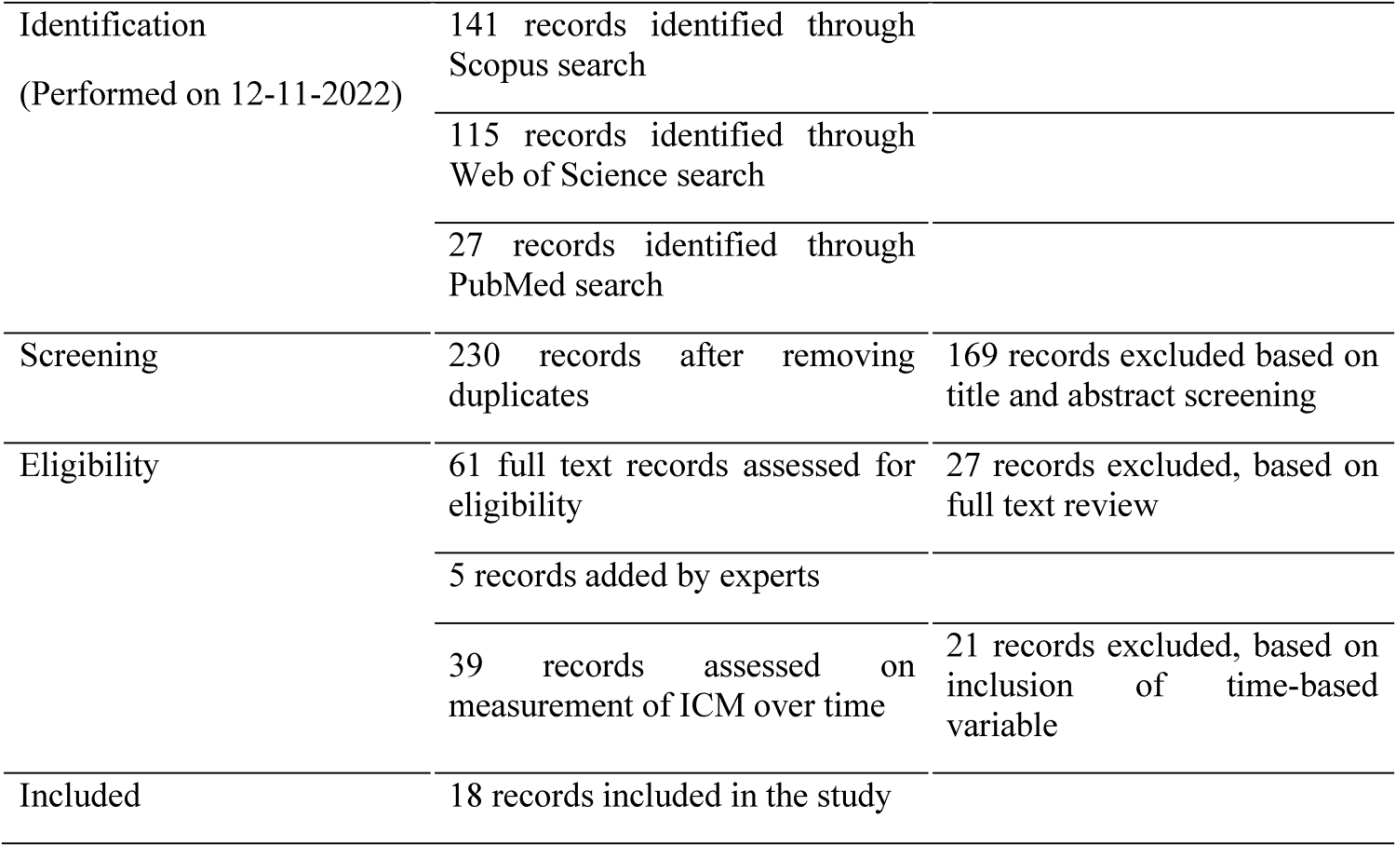
Paper selection process.

Eighteen papers met the inclusion criteria out of the 230 journal papers identified. The selection process and number of records are shown in Figure 1.

Table 2 summarizes key characteristics of these studies, including the authors and year, task context (e.g., office work, driving, aircraft seating), sample size with gender breakdown, task duration, and whether they addressed (ICM) frequency, amplitude, or posture change. The studies span a range of contexts, with participant numbers from 8 to 40 and durations between 18 and 150 min. The remainder of this section presents the results in four parts: an overview of ICM terminology, followed by findings on ICM frequency, amplitude, and posture changes.

**Table 2.** The selected 18 studies.

Authors, Year (reference number)	Context	N (Female + Male)	Duration (minutes)	ICM Frequency	ICM Amplitude	Posture Change
Arippa, Leban, et al., 2022.^18^	Driving (bus)	14M	120	*	*	*
Arippa, Nguyen, et al., 2022.^19^	Office tasks	28 (23F + 5 M)	50 + 10 break (x6)	*	*	*
Baker et al., 2018.^5^	Office tasks	20 (13F + 7 M)	120	*		*
Callaghan et al., 2010.^7^	Driving	24 (12F + 12 M)	60			*
Cascioli et al., 2016.^21^	Office tasks	21 (9F + 12 M)	18	*		
Furugori et al., 2003.^13^	Driving	10 M	120			*
Hermann, 2005.^20^	Driving	12M	150	*		*
Jensen & Bendix, 1992.^16^	Office tasks	10 (5F + 5 M)	Up to 60	*		
Jin et al., 2009.^22^	Driving	8 (2F + 6 M)	100	*		*
Kleine et al., 1999.^26^	Office tasks	9F	60 + 10 break (x3)			*
Maradei et al., 2015.^8^	Driving	18M	90	*		
Na et al., 2005.^23^	Driving	16M	45	*		*
Pinto et al., 2022.^24^	Driving	40 (22F + 18 M)	60	*	*	*
Sammonds et al. 2017.^1^	Driving	10 (4F + 6F)	140	*	*	
Szeto et al., 2002.^25^	Office tasks	16F	50			*
Tanoue et al., 2016.^17^	Office tasks	17 (7F + 10 M)	30	*		
Telfer et al., 2009.^9^	Office tasks	20	120	*	*	
Wang et al., 2021.^30^	Aircraft passenger	19 (6F + 13 M)	50	*		

### ICM terminology

Some of the terminologies used in different papers linked to ICM are shown in Table 3. It varies from “postural change” to “macro-repositioning movements” and “seat fidgets”. These terms also reflect the type of measurement used, and the results that are reported. In this review, “ICM” is used as a general term for human body movements while sitting.

**Table 3.** Overview of terminology of movements during sitting.

Term	Authors, Year [reference number]
Body postures	Callaghan et al., 2010.^7^
Distinct postural change (distinct); Transient postural change; In-Chair Movement (ICM)	Telfer et al., 2009.^9^
Dynamic sitting, pelvic mobility	Tanoue et al., 2016.^17^
In-Chair Movement (ICM)	Cascioli et al., 2016.^21^
In-Chair Movement (ICM)	Wang et al., 2021.^30^
Macro (repositioning) movements. Postural changes	Maradei et al., 2015.^8^
Movement patterns	Jensen & Bendix, 1992.^16^
Neck and shoulder posture, movement excursion	Szeto et al., 2002.^25^
Pelvis movement, Low back angle	Baker et al., 2018.^5^
Postural change	Furugori et al., 2003.^13^
Postural change	Na et al., 2005.^23^
Postural change	Jin et al., 2009.^22^
Postural control	Hermann, 2005.^20^
Postural variation, shifts and fidgets	Pinto et al., 2022.^24^
Seat fidgets and movements	Sammonds et al. 2017.^1^
Sitting posture, Postural parameters	Kleine et al., 1999.^26^
Trunk sway changes	Arippa, Leban, et al., 2022.^18^
Trunk sway. Movement patterns	Arippa, Nguyen, et al., 2022.^19^

### Overall ICM frequency

**Figure 2.**
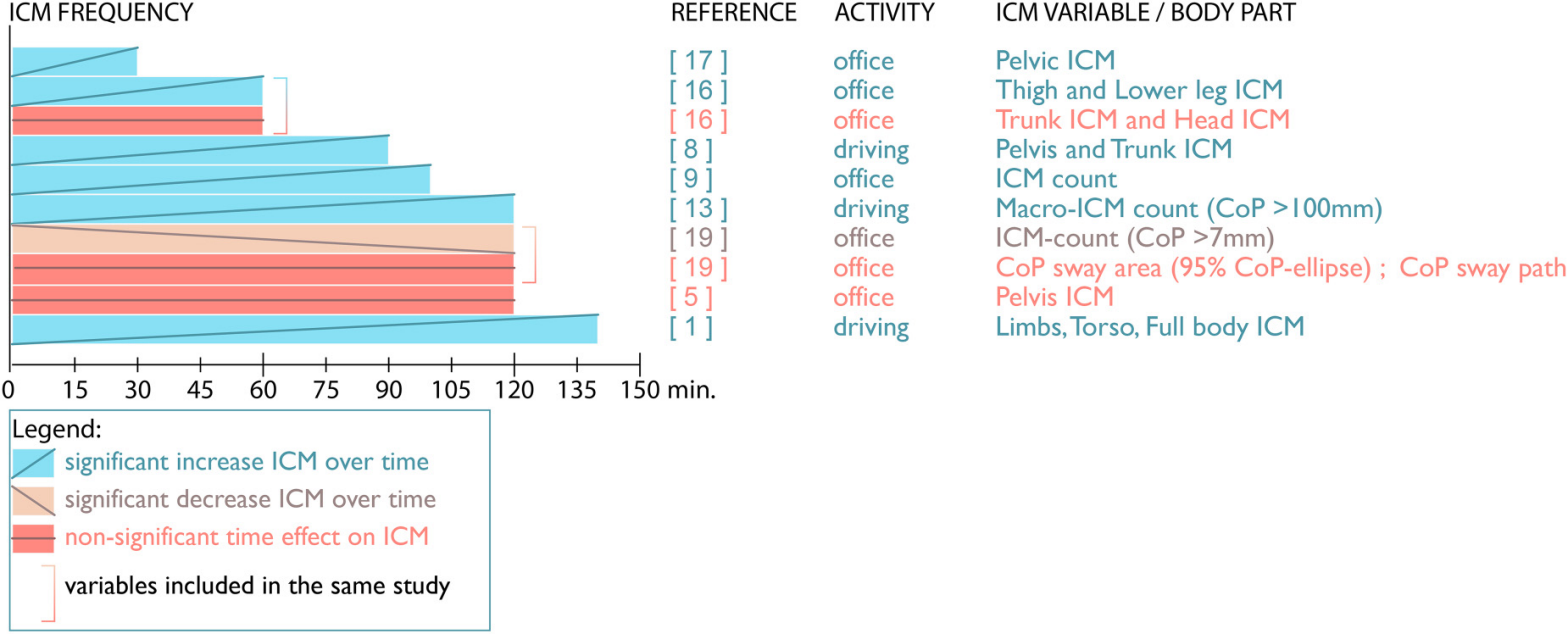
Changes over time in ICM frequency.

Eight out of eighteen papers report on overall time effects on ICM, i.e., the amount of ICM during the total sitting duration (see Figure 2). Six of these eight papers (75%)^1,8,9,13,16,17^ show an increase in ICM frequency over time. One of these, the study of Jensen,^17^ reports an increase in thigh and lower leg ICM but no change in the number of movements of the trunk and head during office work. Two studies (25%) reported different results. The study of Baker et al.^5^ found no change in pelvic ICM during office work. Another study involving office work reported a decrease in ICM frequency over time, as well as no significant changes regarding center of pressure (CoP) variables.^18^

In conclusion, what stands out is that studies examining sitting durations of up to approximately 100 min predominantly show an increase in ICM frequency over time. This is especially true for studies involving an “office work” activity. However, it should be noted that the studies by Arippa et al.^18,19^ and that of Baker et al.,^5^ both involving a two-hour sitting duration, showed either a decrease or a non-significant change in ICM frequency over time. This might suggest less movement over a long duration of office work.

It should also be noted that there are some inconsistencies regarding time effects. Tanoue et al.^17^ found an increase in pelvic ICM over time during a short period, and, similarly, pelvic ICM increased in the 1.5-h study by Maradei et al..^8^ But in a two-hour study, Baker et al.^5^ reported no significant change in pelvic ICM. On the other hand, trunk-ICM showed no significant change in the one-hour study by Jensen and Bendix,^17^ whereas this variable increased in the 1.5-h study by Maradei et al.,^8^ and torso ICM showed significant changes in the 140-min study by Sammonds et al..^1^ This difference in torso ICM may be explained by differences in task or activity, as the study by Jensen et al.^17^ involved office tasks and Sammonds et al.^1^ involved a driving activity. However, regarding pelvic ICM this argument does not hold, as Tanoue et al.^17^ (office activities) and Maradei et al.^8^ (driving activity) showed a similar posture change while the difference occurs between office-based studies by Tanoue et al.^17^ and Baker et al.^5^

A note can be made regarding the ‘sensitivity’ or ‘scale’ of measurement. More specific definitions, such as movements greater than 7 mm,^18^ or precise tracking of the CoP,^19^ were less likely to report significant results. As suggested by several papers, inter-subject differences existed, as well as different movement strategies. Hermann^20^ reported active and passive relief strategies, while distinct and transient postural changes were mentioned by Telfer,^9^ who also suggested that these effects may have overshadowed the sensitivity of ICM measurements.

### ICM frequency intervals

**Figure 3.**
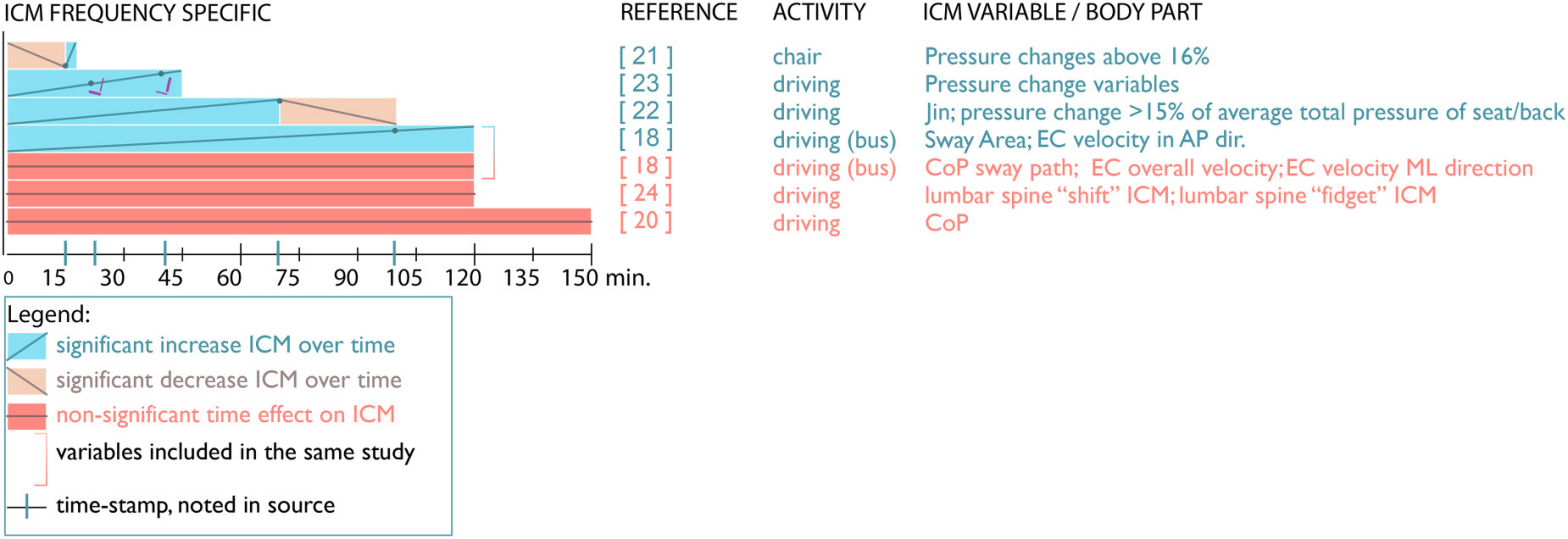
ICM frequency changes between time intervals.

Seven out of 18 papers include comparisons of different time-intervals and/or significance levels at certain timestamps (see Figure 3). One of these papers, the 18-min study by Cascioli et al.,^21^ noted a decrease in ICM frequency at certain intervals. However, in the longer 100-min study by Jin et al.,^22^ a strong increase was noted in ICM frequency between 40 and 70 min, followed by a rapid decline. Both papers used pressure mat data with relatively large threshold values, i.e., pressure changes above 16%,^21^ and 15%,^22^ presumably indicating larger ICM.

The 45-min study by Na et al.^23^ reported a structural increase of ICM frequency, using pressure ratios between seat elements. Interestingly, in their study, ICM in the seat-pan reached significance earlier than ICM in the backrest, at 23 and 41 min, respectively. Moreover, measuring seat-pan pressures, the study of Arippa et al.^19^ reported a structural increase in ICM over 120 min, reaching significance after approximately 100 min of sitting. This moment of significance occurred later than that in the study by Na et al.^23^

On the other hand, three studies included pressure variables that showed no significant changes in ICM frequency over time. The 120-min study by Pinto et al.,^24^ using accelerometers to study both large ICM ‘shifts’ and small ICM ‘fidgets’, found no change in ICM frequency over time. Another 150-min study by Hermann,^20^ showed no increase in ICM frequency, the authors hypothesizing that different ICM strategies overlap over time, masking each other's time effects. Furthermore, several pressure variables in the study by Arippa et al.^19^ involving bus drivers found no significant changes in ICM measures as CoP sway path, pressure change velocity, and pressure changes in sideways direction. The latter study also emphasized the importance of studying not only frequency but also movement direction when distinguishing ICM movement patterns.

### ICM amplitude

**Figure 4.**
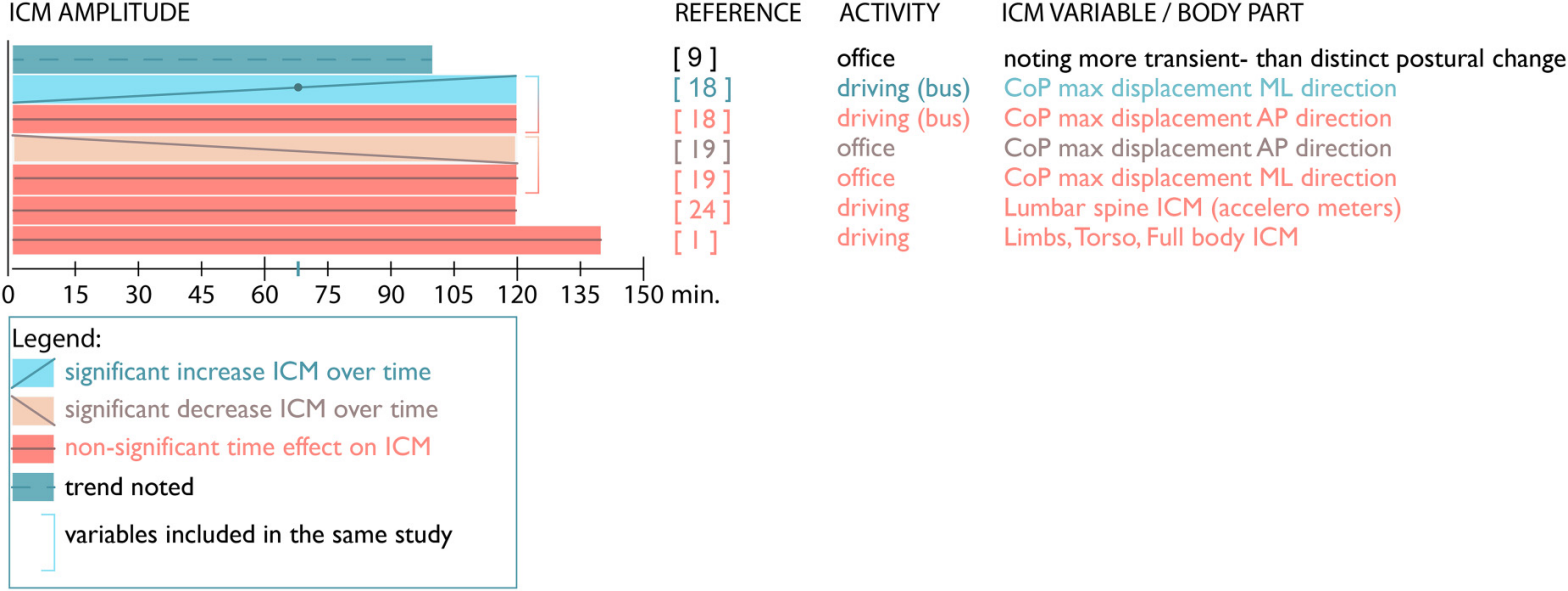
Changes over time in ICM amplitude.

Five of 18 included papers (28%) reported on ICM amplitude over time (see Figure 4). Telfer et al.^9^ noted a trend of more transient posture changes (smaller and shorter ICM) compared to distinct postural changes. This indicates a marginal difference between these two categories. The 120-min study by Arippa et al.^19^ involving bus drivers showed that the maximum displacement, a measure of ICM amplitude, in the medio-lateral (ML) direction increased, especially after 70 min, while the amplitude in the anterior-posterior (AP) direction did not change significantly. However, another study by Arippa et al.^18^ on office workers reported a seemingly opposite effect, with ICM amplitude in the AP direction decreasing over time, thus the amplitude of ICM becoming smaller, while the amplitude in ML direction did not change over time.

On the other hand, two other studies, those by Pinto et al.^24^ and Sammonds et al.,^1^ reported no changes in lumbar spine ICM amplitude over time. Comparing ICM amplitude and frequency here, ICM frequency did not change in the study by Pinto et al.^24^ but did in study by Sammonds et al.^1^ Limb-, torso- and whole-body ICM, distinguished in the latter study, did not change in amplitude over time, although frequency did.

In conclusion, despite a small number of included studies discussing amplitude, results show a mixed image of amplitude over time in relation to movement direction. The seemingly opposite effect regarding changes in the AP and ML directions between studies on office workers and bus drivers, may indicate an effect of the task/activity on ICM amplitude as well.

### Posture change

**Figure 5.**
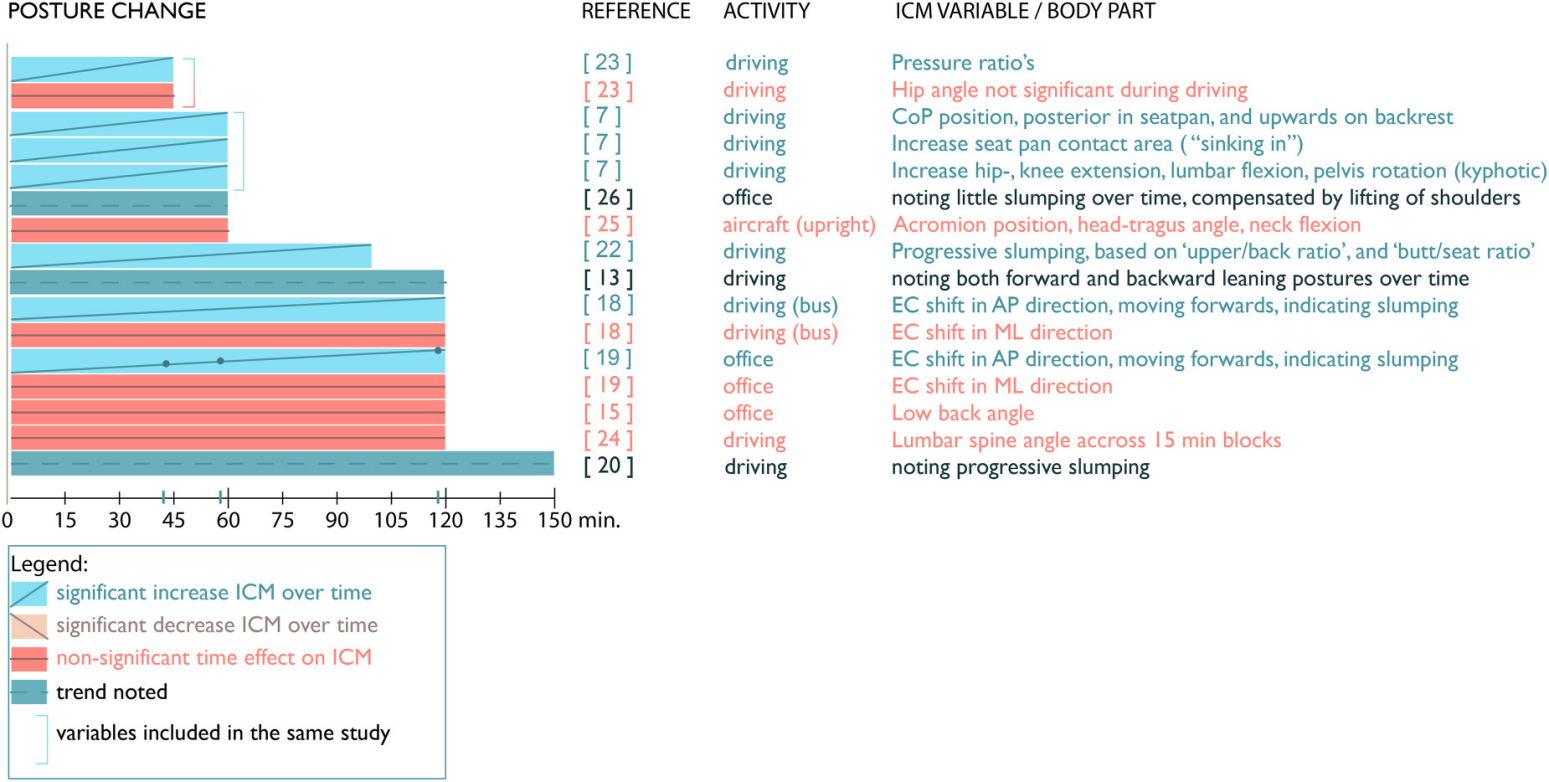
Posture changes over time.

Eleven papers out of 18 (61%) report on posture changes (see Figure 5). Of these eleven, 8 papers (73%) noted ‘slumping’ as a phenomenon over time,^7,13,^^18–20^^,22,23,25^ three of which noted this as a trend.^13,20,25^ Na et al.^23^ reported slumping in their 50-min study, but hip angle, also a measure of posture, did not change over time in their study. Yet, Callaghan et al.^7^ reported increases in hand, hip- and knee joint angles over time, indicating a more kyphotic posture similar to slumping. Slumping was also reported based on decreasing pressure ratios of drivers.^22^ Furthermore, the two-hour studies on bus drivers and office workers showed posture changes in anterior-posterior direction, indicating slumping behavior, where changes in medio-lateral direction were not significant.^18,19^

Three studies reported no significant changes in posture over time. Studying specifically shoulder, neck and head posture, Szeto et al.^25^ reported no posture change over time except for acromion elevation which changed. Low back angle did not change over time in the study by Baker et al.,^5^ and based on accelerometer data, Pinto et al.^24^ found no increase in lumbar spine amplitude or direction of posture shifts.

It should also be noted that studies report different times at which posture changes reach levels of statistical significance. For example, Arippa et al.^18^ noted a decrease of mean pressure in the gluteus region and an increase in the thigh region, similar to the results of Furugori et al.^13^ Interestingly, the mean pressure change reached significance sooner in the thigh region after 57.5 min, than that in the gluteus region after approximately 117.5 min.

Another pattern recognized by some studies, next to slumping, is an occurring asymmetry in pressure profile at times. Related to increasing peak pressure on ischial tuberosities an asymmetric effect was noted by Na^23^ and Hermann et al.^20^

In addition, Callaghan et al.^7^ observed a distinct effect regarding posture change based on pressure area recording, is that the contact area on the seat pan increased. The authors attributed this to a ‘sinking’ of participants into the cushion over time, due to visco-elastic behavior of body tissues and seating foam deformations.

Lastly, three papers comment on trends in posture change over time. Based on marker data, Kleine et al.^26^ described slumping was compensated by a relative lift of the shoulders. Also, Furugori et al.^13^ reported slumping over time based on center of pressure (CoP) position in the backrest and seat pan. Furthermore, their study defined pressure ratio variables indicating the relative amount of pressure on the buttock and upper back areas compared to the total pressure on seat pan and backrest respectively. These ratios showed CoP on the backrest moving downwards and CoP moving forwards on the seat pan, towards the edge of the seat, indicating slumping. However, the authors noted inter-subject differences, some subjects slump over time, while others move to a more leaning forward posture.^13^ Moreover, the 150-min study of Hermann et al.^20^ noted progressive slumping over time.

In summary, posture changes are mentioned by a majority of papers (11 out of 19). Eight out of these eleven papers mention slumping, of which a strong majority (6) concern driving, and two involve an office activity.^18,26^ This suggests that task or activity type influences posture changes, specifically that “driving” tend to promote ‘slumping’ over time. However, some studies observed participants leaning more forward over time, while others reported no significant posture change at all.

## Discussion

### ICM categories

In the reviewed papers, a variety of terms are used to describe in-chair movements. These range from large posture shifts to small fidgets. However, no consensus exists regarding terminology, thresholds, or measurement methods for defining ICM types. Furthermore, the use of both “movement” and “posture change” suggests conceptual inconsistencies or overlaps between these terms. Some terminologies focus more on posture change (e.g., postural change, distinct postural changes, posture shifts), while others report a change in movement parameters such as changes in center of gravity (measured for example by CoP). These changes could be in ICM frequency, amplitude, as well as postural changes. Factors such as participant type, body parts studied, and the activity performed (e.g., driving/office work) should also be considered.

While descriptors such as ICM frequency, amplitude, and posture change were useful for comparing studies within this review, they appear less suitable as the sole basis for defining ICM categories due to the heterogeneity observed within each descriptor. Several studies have proposed the existence of distinct ICM strategies.^9^^18–20^^,26^ Incorporating these strategies into a comprehensive ICM framework could provide a conceptual foundation that integrates multiple ICM descriptors while accommodating individual and contextual variation.

### ICM frequency

Across 18 selected papers, a majority show an overall increase of ICM frequency over time. This is the case for both office and driving activities, but it should be noted that some variables in these activities showed no increase over time. For example, trunk ICM proved to increase during office activity,^16^ driving activities^8^ and similarly, torso ICM increased while driving in the study of Sammonds et al..^1^ Several other papers have stated that ICM are partly driven by tasks e.g., sorting papers on a desk^27,28^ or typing on the keyboard.^29^ Furthermore, pelvic ICM increased in studies by Tanoue et al.^17^ (office activity) and Maradei^8^ (driving), but not in Baker's office-based study.^5^ Thus, tasks or activity alone do not explain these patterns of ICM frequency. Possibly sitting duration, the way the ICM are measured, or person/human characteristics may also influence ICM frequency. Studying these characteristics in relation to ICM should be explored in future research.

### ICM frequency: Stiffening effect in office work

Despite an overall tendency of increasing ICM over time, several studies suggest a stiffening effect or a decrease in ICM over time. Notably, two long-duration studies of two hours observed this effect,^5,19^ which may relate to the nature of office tasks, but deserves more investigation.

Added complexity is given as different studies showing seat elements (e.g., seat pan vs. backrest) reaching significant ICM levels at different times,^18,23^ suggesting that time effects may be body-part- or seat-specific. Seat characteristics, such as foam form^21^ or -hardness,^30^ also influence ICM. For instance, Cascioli et al. (2016) reported that seat characteristics, such as hardness and contouring, were associated with ICM and discomfort, with more ICM observed on hard wooden surfaces compared to straight and contoured foam seats. Wang et al.^30^ also found a correlation between large ICM and increased discomfort. These findings suggest that seats allowing or stimulating greater movement may generate more ICM—a hypothesis worth testing in future studies.

### ICM amplitude: Direction of movement

The fact that Pinto et al.^24^ and Sammonds et al.^1^ both reported no ICM-amplitude changes but differed in ICM-frequency outcomes (no change vs. increased frequency respectively), highlights the need to study how different ICM variables interact, particularly amplitude and frequency. This should be linked to seat shapes and environmental constraints, as seating design and environment can restrict certain movements. For instance, lateral trunk movement may be easier to perform in office chairs than in car seats with pronounced lateral supports.

Also, the studies by Arippa et al.^18,19^ showed different direction-specific results; anterior-posterior (AP) amplitude decreased for office workers but increased in medio-lateral (ML) direction for bus drivers. This supports the importance of analyzing movement direction, not just frequency, when characterizing ICM.

### Posture change: Slumping behavior and postural strategies

Slumping over time is a posture change observed by most papers, particularly those involving driving. Defining ‘slumping’ is challenging, as illustrated by contrasting findings between Furugori et al.^13^ and Callaghan et al.,^7^ a discrepancy also acknowledged by Callaghan et al.^7^ While both studies report slumping, Callaghan et al.^7^ observed CoP shifting toward the back of the seat pan and upward in the backrest, which contradicts kyphotic posture findings from Furugori et al.^13^ This inconsistency underscores the need for clearer definitions and measurement approaches for posture strategies.

### Posture change: Human-body effects

Although three papers reported no significant posture changes over time, it should be noted that all three papers focused on specific body parts. Szeto et al.^25^ studied shoulder and head-tragus positions, Pinto et al.^24^ distinguished ‘posture shift’ as a change from back flexion- to extension or vice versa, and “fidgets” as changes in lumbar angle, based on earlier ICM categorization by Dunk and Callaghan.^31^ Similarly, low back angle was also specifically studied by Baker et al.^5^ This may indicate that, while analyzing posture change, focusing on individual body segments in ICM analysis may be less effective.

Moreover, the fact that posture changes occur at different timestamps across body parts suggests distinct postural strategies, possibly related to human-, seat- or activity characteristics. These different strategies and their associated human-body responses may be a ‘direct’ feedback loop of ‘adjusting to or coping with’ discomfort, consistent with the comfort model of Vink & Hallbeck.^2^ Therefore, recognizing these posture changes may offer insights into the relationship between sitting strategies and discomfort.

### Limitations

Although the PRISMA method for literature review requires careful selection of search terms and databases, several terms were inherently excluded as considered out of scope for this paper.

For example, in relation to physiology, relationships have been established between prolonged sitting and muscle fatigue,^32^ and ICM and spinal loading and heart rate variability.^33^ A relation between obstructed blood flow in the skin, known as skin ischemia, has also been hypothesized as a physiological symptom of discomfort,^21^ and ICM has been hypothesized as a mechanism for pressure relief on ischial tuberosities.^20^ Furthermore, Sharafkhani (2021)^34^ noted that subjects verbally described discomfort in terms of joint stiffness and muscle fatigue, indicating a correlation between discomfort perception and physiology. Therefore, studies on physiological parameters in relation to ICM and discomfort deserve further attention in further research on ICM.

Seat characteristics have also proven to be a factor contributing to (dis)comfort, such as shapes^30^ or stiffness of cushions.^21^ Additionally, dynamic seating, whether actively or passively moving^3^ was not included in this review. However, such seating systems have been associated with increased ICM and positive effects on the body, including reduced muscle stiffness^35^ and lower discomfort.^3,36^ Further research on ICM should therefore also consider dynamic seating, particularly how seat characteristics can facilitate^27,37^ and/or stimulate ICM^35^ and their possible correlations with discomfort perceptions.

Lastly, the present paper includes studies involving different activities such as driving and office tasks. Although this provides a broader overview and shows correlations between these activities on ICM variables, the findings should be interpreted as indicative trends requiring further investigation. Contextual and environmental factors may affect human behavior during activities, which in turn may affect ICM and discomfort perception.^38^ Therefore, the generalizability of these trends requires careful consideration when establishing correlations between activities and ICM in further research.

## Conclusion

The aim of this review was twofold: first, to examine what types of in-chair movements (ICM) have been categorized across literature, and second, to evaluate reported time-related ICM patterns.

Regarding the first objective, the review revealed inconsistencies in terminology and used measurement methods, with overlap between concepts of “movement” and “posture change”. Developing an ICM framework which includes movement strategies and movement descriptors may therefore facilitate greater consistency in future ICM research.

In relation to time-trends, despite considerable heterogeneity in reported outcomes, most studies reported an increase in ICM frequency as sitting duration progressed, as well as posture changes such as slumping. However, some studies did not observe these patterns, including studies showing reduced movement or a “stiffening effect” in longer tasks, particularly in office contexts. These mixed findings indicate that ICM patterns are not solely time-dependent but are shaped by seat characteristics, task demands, and human factors.

Across both objectives, a consistent theme is the link between ICM strategies and discomfort. Several studies reported correlations between ICM parameters and overall or local discomfort scores. Nevertheless, the mechanisms linking ICM strategies to perceived discomfort remain unclear.

Finally, the findings highlight future opportunities to investigate dynamic sitting. Seats that enable or encourage movement may not only alter ICM patterns but could also contribute to mitigating discomfort during prolonged sitting. Incorporating such seating approaches into ICM research, alongside the development of a comprehensive ICM framework, offers promising directions for future work. Together, these efforts can improve understanding of how humans adapt their sitting behavior through ICM strategies and how these adaptations relate to comfort and health.
